# Suppression of c-Kit signaling induces adult neurogenesis in the mouse intestine after myenteric plexus ablation with benzalkonium chloride

**DOI:** 10.1038/srep32100

**Published:** 2016-08-30

**Authors:** Hiromi Tamada, Hiroshi Kiyama

**Affiliations:** 1 Department of Functional Anatomy & Neuroscience, Nagoya University, Graduate School of Medicine, 65 Tsurumaicho, Showaku, Nagoya 466-8550 Japan

## Abstract

Adult neurogenesis rarely occurs in the enteric nervous system (ENS). In this study, we demonstrated that, after intestinal myenteric plexus (MP) ablation with benzalkonium chloride (BAC), adult neurogenesis in the ENS was significantly induced in *c-kit* loss-of-function mutant mice (*W/W*^*v*^). Almost all neurons and fibers in the MP disappeared after BAC treatment. However, 1 week after ablation, substantial penetration of nerve fibers from the non-damaged area was observed in the MP, longitudinal muscle and subserosal layers in both wildtype and *W/W*^*v*^ mice. Two weeks after BAC treatment, in addition to the penetrating fibers, a substantial number of ectopic neurons appeared in the subserosal and longitudinal muscle layers of *W/W*^*v*^ mice, whereas only a few ectopic neurons appeared in wildtype mice. Such ectopic neurons expressed either excitatory or inhibitory intrinsic motor neuron markers and formed ganglion-like structures, including glial cells, synaptic vesicles and basal lamina. Furthermore, oral administration of imatinib, an inhibitor of c-Kit and an anticancer agent for gastrointestinal stromal tumors, markedly induced appearance of ectopic neurons after BAC treatment, even in wildtype mice. These results suggest that adult neurogenesis in the ENS is negatively regulated by c-Kit signaling *in vivo*.

The enteric nervous system (ENS), categorized as the third autonomic nervous system, forms huge neuronal networks along the gastrointestinal tract^1^. Two main nerve plexuses, the myenteric plexus (MP) and the submucosal plexus (SP), exist in the ENS; each of these consists of enteric neurons, enteric glial cells, and a mesh of nerve bundles. These intrinsic nervous systems control intestinal behavior autonomously. In addition, exogenous autonomic and sensory inputs modulate the nervous system. The ENS is crucial for normal gastrointestinal functions such as digestion and absorption and is also susceptible to injury^2^. Therefore, more understanding of how to repair the ENS after injury or surgery is needed.

Adult neurogenesis in the central nervous system (CNS) has been well described^3^, but adult neurogenesis in the ENS remains elusive. Though ENS neurogenesis *in vivo* is rarely observed under normal conditions^4^,^5^, the appearance of a few neurons was reported following injury or benzalkonium chloride (BAC) treatment, the latter ablating neurons^6^,^7^. More recently, 5-HT_4_ receptor-mediated signaling was implicated in adult neurogenesis in the ENS^2^,^8^,^9^. The involvement of 5-HT_4_ receptors in adult neurogenesis of the ENS was confirmed using 5-HT_4_ receptor knockout mice^2^ and a 5-HT_4_ receptor agonist^2^,^8^,^9^. These studies demonstrated that progenitor cells capable of undergoing neurogenesis exist in the adult intestine, but that neurogenesis does not occur under steady-state conditions^10^. In addition to 5-HT_4_-mediated mechanisms, other unknown mechanisms that trigger conversion of silent progenitors into a neurogenic state in the adult ENS are likely to exist.

c-Kit, a receptor tyrosine kinase that binds to stem cell factors, is expressed on the surface of interstitial cells of Cajal (ICC) in the intestine as well as on hematopoietic stem cells and mast cells. In the gastrointestinal tract, c-Kit was predominantly expressed in the ICC and this was shown to be necessary for ICC development and maintenance^11^,^12^,^13^. Gain-of-function mutations of c-Kit exist in the gastrointestinal stromal tumor (GISTs), the most common mesenchymal tumors in the human gastrointestinal tract. Gain-of-function mutations of c-Kit were identified in more than 80–90% of GISTs^14^, and GISTs were, thus, considered to originate from ICCs or their precursor cells^15^. In contrast, a loss-of-function c-Kit mutation exists in the *W/W*^*v*^mouse, leading to several gastrointestinal disorders^11^,^16^. The *c-kit* gene is allelic with the murine white-spotting locus (*W*) and a heterozygote for two distinct mutations occurs in the *W/W*^*v*^*strain*. These are a deletion in the c-Kit transmembrane domain (*W*) and a point mutation in the c-Kit kinase domain (*W*^*v*^)^17^,^18^,^19^. In this mutant mouse, abnormal localization of ICC occurs^20^,^21^. For example, ICC associated with the myenteric plexus in the small intestine disappeared, suggesting c-Kit dependent differentiation and/or maintenance of ICC in those regions^16^. We demonstrated the existence of a c-Kit-negative ICC in the subserosal layer of the colon in *W/W*^*v*^ mice^22^. In a series of studies using *W/W*^*v*^ mice, we examined effects of BAC ablation on intestinal neurons. The surfactant BAC elicits neuronal death in the MP^23^. In this study, we noticed, in addition to intestinal neuronal ablation, the appearance of ectopic neurons in the longitudinal muscle (LM) and the subserosal (SS) layers in *W/W*^*v*^ mice treated with BAC. To investigate whether the appearance of ectopic neurons was incidental or, instead, associated with a certain condition, we here examined the conditions leading to appearance of these neurons and addressed potential mechanisms underlying their appearance.

## Results

### Appearance of NADPH-positive neurons after BAC treatment

**Figure 1:**
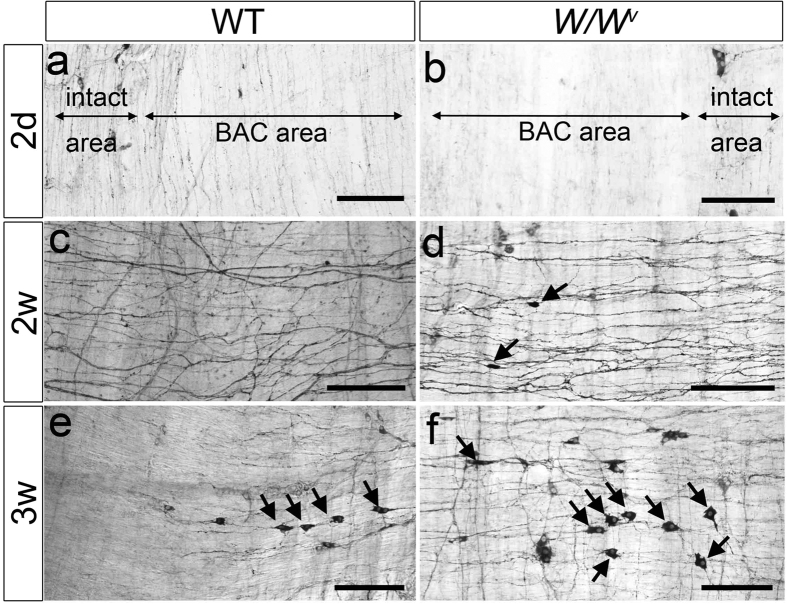
NADPH-diaphorase staining using whole-mount preparations after BAC treatment. **(a,b)** Two days after BAC treatment. NADPH-diaphorase-positive neurons and fibers in the myenteric plexus were no longer observed in the treated areas; however, neurons remained in the intact, non-BAC treated, areas. ((**a**) wildtype, n = 3, (**b**) *W/W*^*v*^, n = 3). **(c,d)** Two weeks after BAC treatment. Numerous regenerating nerve fibers were observed in the longitudinal muscle layer of both wildtype (n = 3) (**c**) and *W/W*^*v*^ (n = 3) mice (**d**). In *W/W*^*v*^ mice (**d**) a few NADPH+ cells (arrows) were visible, but not in the wildtype mice (**c**). **(e,f)** Three weeks after BAC treatment. A few NADPH+ cells were detected in wildtype mice (n = 7) (arrows in **e**). *W/W*^*v*^ had abundant NADPH+ cells (n = 8) (arrows in **f**). Scale bar, 200 μm (**a–f**).

Two days after BAC treatment, neurons and nerve fibers in the MP, identified by NADPH-diaphorase staining, had disappeared in wildtype mice (Fig. 1a). PGP9.5 staining also confirmed complete loss of neurons in the MP after BAC treatment (data not shown). This ablation of neurons in the MP was similarly observed in *W/W*^*v*^ mice (Fig. 1b). One week after BAC treatment, newly appearing NADPH+ nerves were observed in the LM, SS and MP. The number of these nerve fibers was increased at 2 weeks after BAC treatment in both wildtype and *W/W*^*v*^ mice (Fig. 1c,d). In the normal intestine, there were apparently few nerve fibers in the LM and the SS, whereas, after BAC treatment, a significantly greater number of fibers were identified in the LM and SS of both wildtype and *W/W*^*v*^ mice. Intriguingly a few NADPH+ cells were evident in the LM and SS at 2 weeks after BAC treatment in *W/W*^*v*^ mice (Fig. 1d, arrows), whereas no NADPH+ neurons were identified in any layers in wildtype mice. At 3 weeks after BAC induced injury, a dramatic increase in NADPH+ cell number was found in *W/W*^*v*^ mice, whereas only a few positive neurons were observed in wildtypes (Fig. 1e,f).

### Morphological analysis of newly appearing NADPH-positive cells

**Figure 2:**
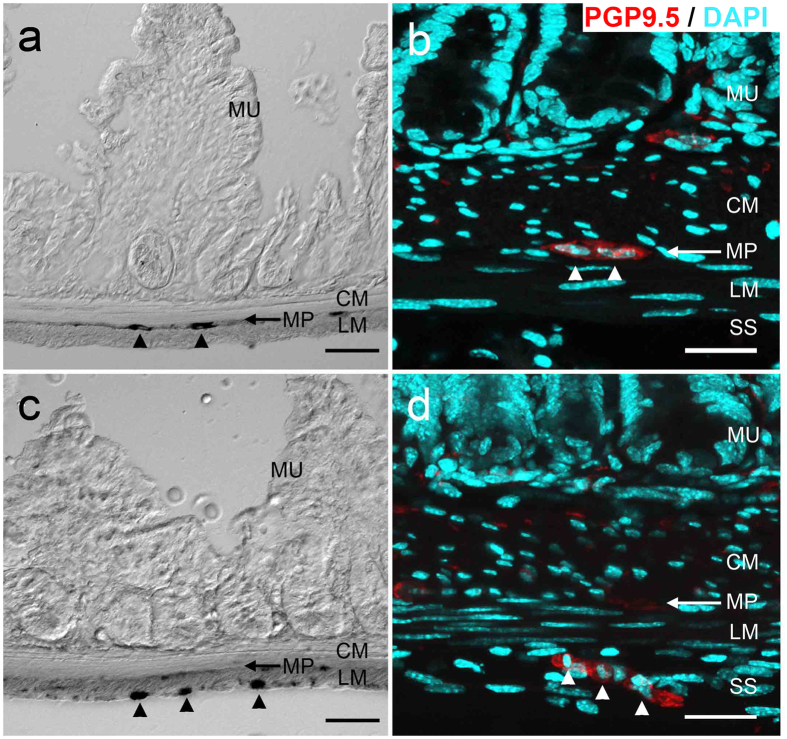
Location of NADPH-positive neurons. Three weeks after BAC treatment, newly appearing neurons in *W/W*^*v*^ mice (n = 3) were identified by NADPH and PGP9.5 staining using cross sections (**c**,**d**), and compared with staining in untreated tissue (n = 3) (**a**,**b**). **(a,b)** The myenteric plexus contained NADPH+ cells (**a)** and PGP9.5+ cells ((**b)**, arrowheads). There were no NADPH+ neuron-like cells or PGP9.5+ cells beyond the myenteric plexus. **(c)** In samples obtained 3 weeks after BAC treatment, new NADPH+ cells (arrowsheads) were located in the subserosal (SS) and longitudinal muscle (LM) layers, areas where neurons do not normally exist. **(d)** PGP9.5-positive neurons (arrowheads) located in the subserosal layer. PGP9.5 (red), DAPI (blue), MU, mucosa; CM, circular muscle layer; MP, Myenteric plexus; LM, longitudinal muscle layer; SS, subserosal layer; Scale bar, 100 μm (**a,c**) or 30 μm (**b,d**).

**Figure 3:**
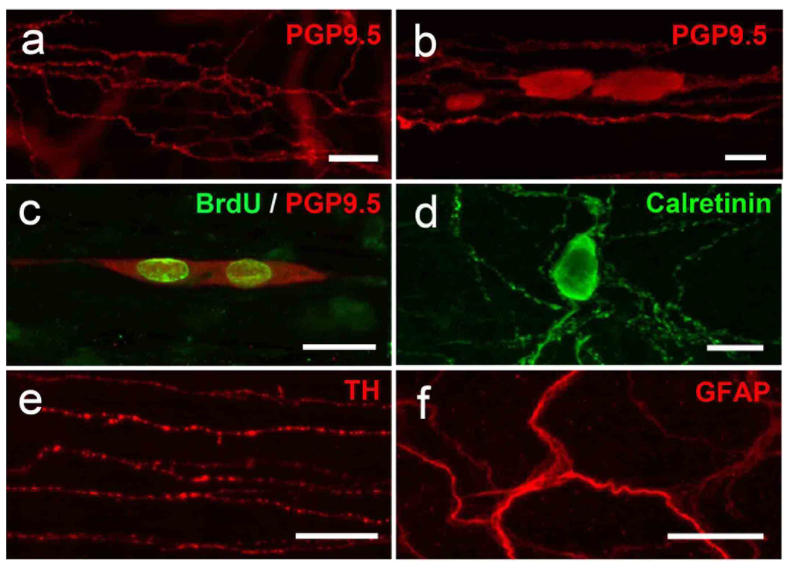
Characteristics of ectopic neurons. Whole-mount preparations from *W/W*^*v*^ mice 3 weeks after BAC treatment were used to characterize ectopic neurons by immunohistochemistry. **(a)** Numerous PGP9.5-positive nerve fibers were observed in the LM. Scale bar, 50 μm. **(b)** PGP9.5-positive cells together with fibers were detected in the SS. Scale bar, 20 μm. (n = 10 for (**a,b**)). **(c)** Most ectopically appearing PGP9.5-positive neurons were also BrdU-positive in the SS (n = 5). Scale bar, 50 μm. **(d)** Calretinin immunoreactive cells and fibers were observed in the SS (n = 5). Scale bar, 20 μm. **(e)** Some regenerating nerve fibers were TH-positive in the SS (n = 4). Scale bar, 50 μm. **(f)** GFAP-positive cells and processes were also detected in the SS (n = 7). Scale bar, 50 μm.

In the normal ileum, enteric neurons are distributed in myenteric plexus (MP) and submucosal plexus (SP) (Fig. 2a,b). Because the newly appearing NADPH-positive cells seemed to be located in the outer layers rather than in the MP, we examined newly appearing cells in tissue cross-sections. We found that most of the NADPH-positive cells were located in the LM and SS (Fig. 2c). Staining with an antibody against PGP9.5, a marker for pan-neuronal structures, was used to evaluate whether the newly appeared cells were neuronal cells. Simultaneously, we stained with DAPI as a nuclear marker to identify layers and cells. As shown in Fig. 2d, the morphology of nuclei could be used as a marker to determine circular muscle (CM) layer cells, where nuclei appeared round, or LM layer cells, where nuclei appeared longer. PGP9.5-positive cells were located in the LM and SS, where NADPH-positive cells were located (Fig. 2c,d). Using flat-mount preparations, we confirmed that, after BAC treatment, newly appearing cells and fibers in the outer layers were also PGP9.5-positive and that the localization was comparable with that of NADPH-positive cells (Fig. 3a,b). This suggested that the newly appearing cells in the LM and SS were ectopic neurons.

To determine whether these ectopic neurons were migrating or proliferating cells, animals were treated with BrdU for 3 weeks after BAC treatment. BrdU-positive ectopic neurons, which were also PGP9.5-positive, were identified in the SS or LM, indicating that these ectopic neurons were newly born after BAC ablation (Fig. 3c).

The neural plexus known as the MP is composed of intrinsic excitatory and inhibitory motor neurons and glial cells. This led us to examine the characterization of the newly appeared neurons. Because NADPH+ neurons are usually classified as intrinsic inhibitory motor neurons, we investigated the existence of intrinsic excitatory motor neurons, whose processes are elongated into the LM from the MP^24^, using calretinin as a marker. Some ectopic cells had calretinin-positive immunoreactivity at 3 weeks after BAC treatment (Fig. 3d). In the normal intestine, TH positive fibers, which are sympathetic and innervate extrinsically, were observed, though there were relatively few TH positive fibers in LM and SS. After BAC treatment, the TH positive fibers temporarily disappeared because of the BAC-induced neuronal damage. At 3 weeks after BAC treatment, TH positive fibers reappeared in the LM and SS, which indicated an invasion of extrinsic sympathetic nerves (Fig. 3e). GFAP-positive glial cells, which disappeared after BAC treatment, were also identified in these layers, suggesting that migrating or newly generated glial cells were involved in this ectopic neuronal plexus (Fig. 3f).

**Figure 4:**
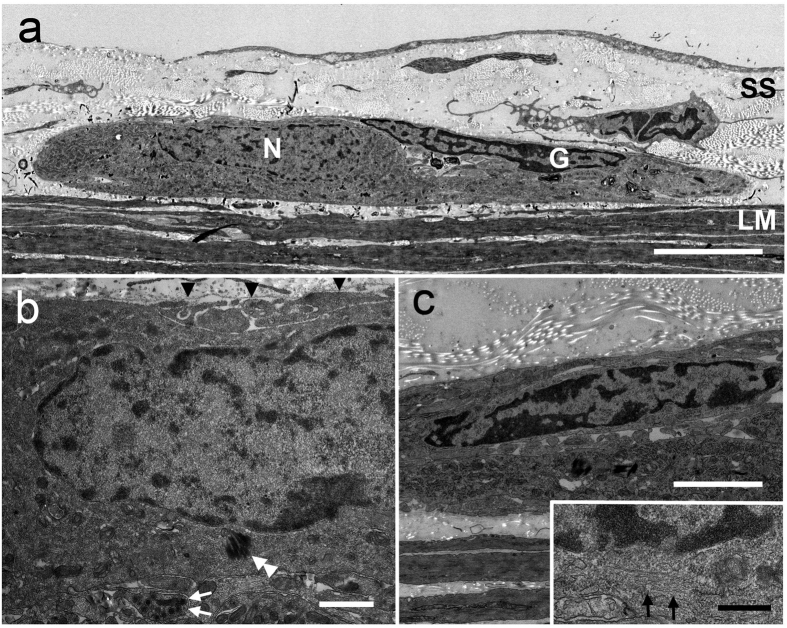
Electron microscopy of ectopic ganglia in the SS. BAC treated tissue from *W/W*^*v*^ mice was weakly stained with NADPH-diaphorase to detect neurons and tissue was then processed for electron microscopy (EM) (n = 3). **(a)** Low power EM magnification. A typical ganglion containing both neurons (N) and glia (G) in the SS is shown. SS, subserosal layer; LM, longitudinal muscle layer. Scale bar, 5 μm. **(b)** Enteric neurons in ectopic ganglia had large and few heterochromatin nuclei. Osmium black (double arrowheads) staining in the cytosol indicates NADPH-diaphorase activity. Basal lamina (arrowheads) surrounded the ganglion. Nerve bundles containing synaptic vesicles (arrows) were also observed in the ganglion. Scale bar, 1 μm. **(c)** Enteric glial cells with heterochromatin-rich nuclei were detected in the ectopic ganglion. The 10-nm filaments in the cytosol were also observed in glial cells (inset, arrows). Scale bar, 2 μm; inset, 500 nm.

After identifying the ectopic NADPH-diaphorase-positive cells by light microscopy, we processed the same sections for electron microscopy. These experiments revealed ganglion-like structures in the SS layer (Fig. 4a). Two types of cells, exhibiting different cytosolic and nuclear characteristics, were identified in ectopic ganglia that were surrounded by the basal lamina (Fig. 4b arrowheads). One cell type had a large nucleus with few heterochromatin and osmium black deposits caused by NADPH-diaphorase immunoreactivity, indicating that they were NADPH-positive neurons (Fig. 4b, double arrowheads). The other cell type had a distinct electron dense cytosol and heterochromatin-rich nuclei, which indicated that they were enteric glial cells (Fig. 4c). These glial cells also showed 10-nm gliofilaments in the cytoplasm (Fig. 4c, inset). In addition, nerve bundles with synaptic vesicles (Fig. 4b arrows) were observed in the ectopic ganglion. From these findings, we concluded that ectopic ganglia contained similar components to ordinary enteric ganglia seen in the MP.

### The number of ectopic NADPH-positive cells in wildtype and *W/W* ^*v*^ mice

**Figure 5:**
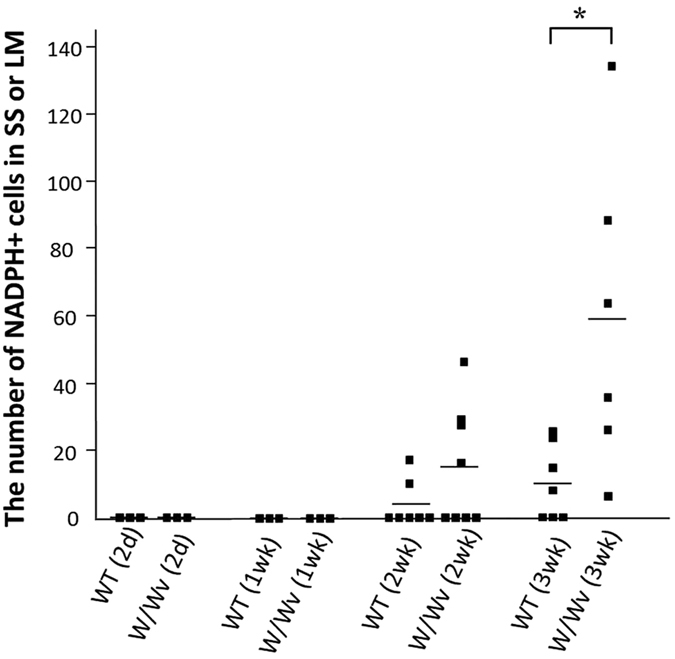
Numbers of ectopic NADPH-positive cells in the SS and LM. Each plot (boxes) indicates the number of NADPH+ cells in the subserosal (SS) and longitudinal muscle (LM) layers per damaged area, at each time point after BAC treatment (2 days–3 weeks). The means (bars) are also plotted at each time point for both wildtype (W) and *W/W*^*v*^ mice. No NADPH+ cells were observed in either wildtype or *W/W*^*v*^ derived samples during the first week of BAC treatment (wildtype 2 days, n = 3, *W/W*^*v*^ 2 days, n = 3, wildtype 1 week, n = 3, *W/W*^*v*^ 1 week, n = 3). On weeks 2 and 3 after BAC treatment, the average number of cells was 3.9 ± 2.4 (mean ± SEM, n = 7) and 10.6 ± 3.9 (n = 7), respectively, per damaged area for wildtype mice. On weeks 2 and 3 after BAC treatment, the average number of cells was 15.1 ± 6.0 (n = 8) and 59.2 ± 17.6 (n = 6), respectively, for *W/W*^*v*^ mice. The increase in NADPH-positive cells in *W/W*^*v*^ mice compared with that in wildtype mice was statistically significant at 3 weeks after treatment (*P < 0.05).

We next counted NADPH-positive neurons. Because BAC treatment was applied to one restricted area of the intestine, each mouse had only one damaged intestinal area. We counted NADPH-positive cells contained in the entire BAC treated area. There were fewer NADPH-positive cells than there were PGP9.5-positive neurons. This was most likely because of the additional calretinin-positive neurons, generated in parallel after BAC treatment. Because individual neurons and nerve fibers could be identified much more clearly with NADPH staining, compared with PGP9.5 immunostaining, particularly in flat-mount preparations, we counted NADPH-positive neurons. The disappearance of PGP9.5-positive staining after BAC treatment and its appearance beginning 1 week after BAC treatment occurred in parallel with the changes in NADPH staining. The numbers of ectopically appearing NADPH-positive neurons in wildtype and *W/W*^*v*^ mice were different (Fig. 5). More neurons were observed in *W/W*^*v*^ mice than in wildtypes after neuronal ablation by BAC treatment. At 1 week after the ablation, no NADPH-positive neurons were seen in either wildtype (n = 3) or *W/W*^*v*^ mice (n = 3). At 2 weeks after the ablation, 3.9 ± 2.4 cells (mean ± SEM) were detected in the damaged area (approximately 2 × 0.5 cm^2^) of wildtype mice (n = 7), compared with 15.1 ± 6.0 cells in *W/W*^*v*^ mice (n = 8) (Fig. 5). At 3 weeks after the ablation, 10.6 ± 3.9 cells appeared in wildtypes (n = 7), whereas there were 59.2 ± 17.6 cells in *W/W*^*v*^ mice (n = 6). Though the number of NADPH-positive neurons varied among animals, there were significantly more NADPH-positive cells in *W/W*^*v*^ mice than in wildtype mice at 3 weeks after BAC (*P* < 0.05; Mann–Whitney U test).

### Imatinib administration to wildtype mice

**Figure 6:**
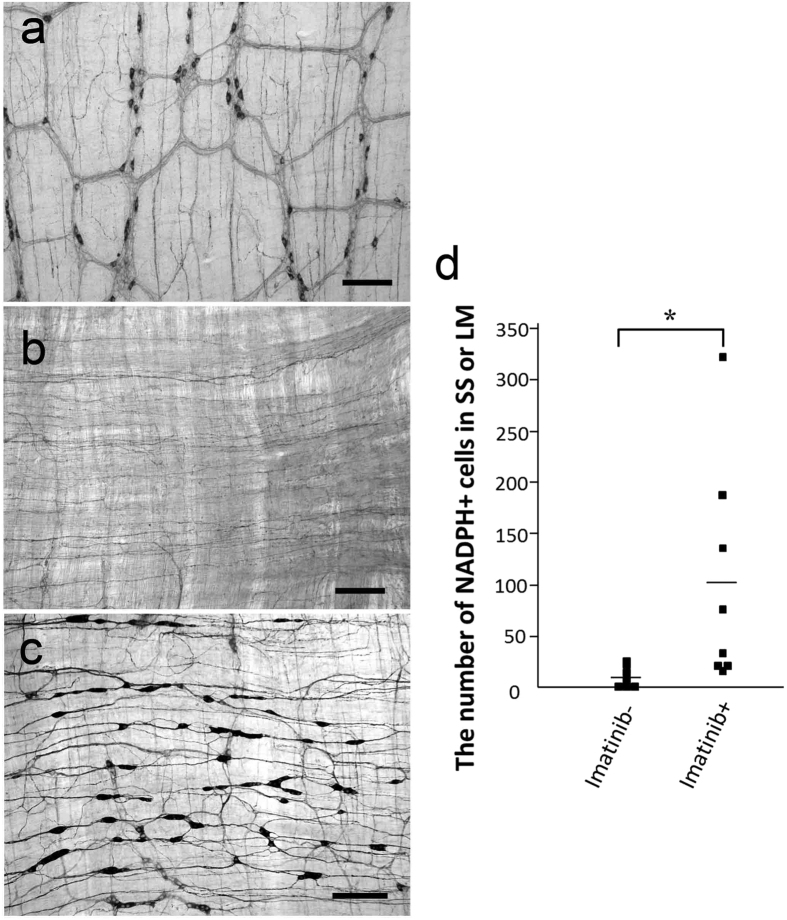
Imatinib induced appearance of NADPH-positive cells in the SS and LM of wildtype mice. **(a)** The normal ileum without BAC ablation showed networks of myenteric plexus. There were no neurons beyond the myenteric plexus layer. Ectopic NADPH-positive cells appeared in wildtype mice after BAC treatment in the presence of vehicle (**b**) or imatinib (**c**). Scale bar, 200 μm. (**d**) The number of NADPH-positive cells following treatment with vehicle or imatinib in wildtype mice (boxes) 3 weeks after BAC treatment. The average number of ectopic NADPH-positive cells in wildtype mice treated with vehicle and imatinib were 10.6 ± 3.9 (n = 7) and 103.4 ± 36.1 (n = 8), respectively (*P < 0.01).

To further address involvement of c-Kit, which was not expressed in *W/W*^*v*^ mice, we used the c-Kit inhibitor imatinib, a treatment for GIST in humans. Imatinib was administered orally for 3 weeks (every 2 days) to wildtype mice. Imatinib treatment substantially increased numbers of NADPH-positive neurons in the LM and SS of wildtype mice after BAC treatment (Fig. 6b,c). These neurons were not observed in the normal ileum (Fig. 6a). Though the variation in numbers of NADPH-positive cells among animals was higher with imatinib, the average number of newly identified neurons increased almost 10 fold (untreated: 10.6 ± 3.9 cells, n = 7 vs. imatinib treated: 103.4 ± 36.1 cells, n = 8) (*P* < 0.01; Mann–Whitney U test) (Fig. 6d).

## Discussion

**Figure 7:**
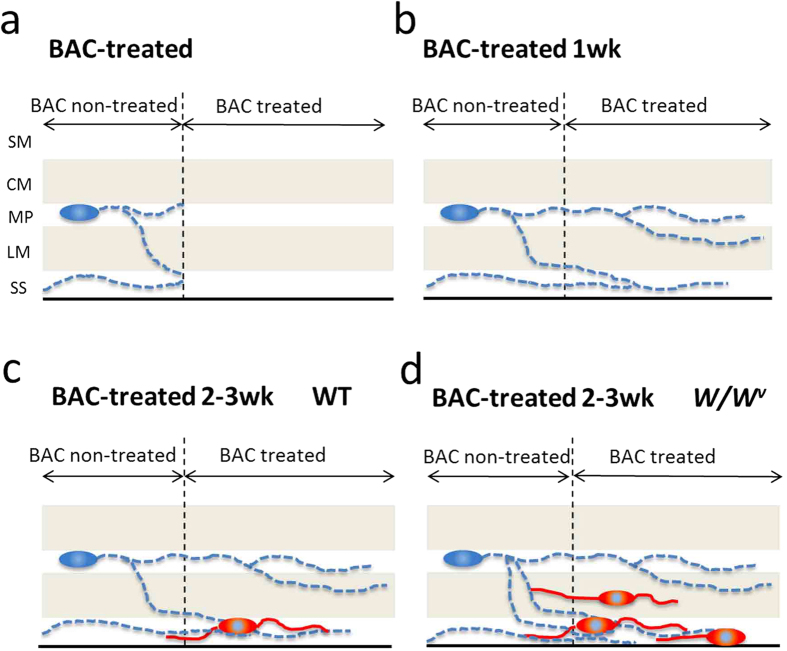
Schematic summary. After BAC treatment, neurons and nerve fibers observed in the MP, LM and SS (blue) disappeared **(a)** and subsequent infiltration of nerves from intact areas was observed 1 week later **(b)**. Beginning at 2 weeks, new neurons and their associated fibers appeared ectopically (red) in the LM and SS of both wildtype **(c)** and *W/W*^*v*^ **(d)** mice. However, the number of new neurons was markedly greater in *W/W*^*v*^ than in wildtype mice.

The occurrence of adult neurogenesis in the ENS under physiological and pathological conditions has been debated. A consensus opinion would include that progenitor cells, capable of undergoing neurogenesis in the adult gut, exist and that neurogenesis does not occur under steady-state conditions^6^,^7^,^10^. However, adult neurogenesis was observed under conditions of injury and/or in a 5-HT_4_-mediated manner^2^,^7^,^10^. In addition to those previous reports, our findings suggested a novel mechanism underlying adult neurogenesis in the ENS, involving suppression of c-Kit signaling. The present results are summarized in Fig. 7.

In previous studies on ENS neurogenesis after neuronal ablation with BAC, limited neurogenesis was observed at 1–3 months after BAC treatment^6^,^7^. These investigators demonstrated that even wildtype mice had the potential to generate new neurons after BAC treatment. Our study confirmed neurogenesis in wildtype mice. Therefore, tissue damage is a critical factor promoting ENS neurogenesis in wildtype mice. BAC treatment from the subserosal side damaged the tissue through to the MP layer. The submucosa and adjacent circular muscle layers, where BAC did not penetrate, appeared intact. This may be one reason why neurons in the MP disappeared, whereas those in the submucosa layer survived. Though smooth muscle cells in the LM were nonspecifically damaged, they were apparently repairable immediately after damage, appearing normal beginning 1 week after BAC treatment^23^. It is therefore likely that the injured smooth muscle cells or other cells located from the LM to the outer layers released unknown factors that trigger neurogenesis of progenitor cells and/or recruit progenitor cells from other areas in response to injury. This may also explain why we observed newly generated neurons and glia ectopically localized in the LM and outer layers.

Our study, as well as previous ones, demonstrated relatively rapid re-innervation of nerve fibers in the ablated area after BAC treatment^6^,^10^. As both TH-positive and -negative nerve fibers were observed in this region, sympathetic fibers from outside the intestine were also, simultaneously, re-innervated in the area. Most of the newly appearing fibers were traced from the MP of the intact area and their numbers were comparable in wildtype and *W/W*^*v*^ mice. This suggested that both enteric neurons located in the intestine and sympathetic fibers from outside of the intestine have high potential for neurite regeneration, even in wildtype mice. Following neurite re-innervation, ectopic neurons and ectopic ganglia, including both excitatory and inhibitory neurons as well as glial cells, appeared. Though our study did not address the origin of these newly identified neurons after BAC treatment, some reports described the presence of potent precursor cells, namely, enteric glial cells (EGCs), neural crest-derived stem cells (NCSCs) and Schwann cell precursors (SCPs). Among these, EGCs shared many phenotypic properties with NCSCs^7^,^10^. Several reports addressed the existence of NCSCs, capable of differentiating into neurons *in vitro*, in the adult gut^25^. However, a major impediment to addressing this issue has been the lack of convincing markers to identify NCSCs in the adult gut *in vivo*. While analysis of NCSCs has been performed, recent genetic approaches using fate mapping studies elucidated EGCs as a source of adult neurogenesis. EGCs showed multipotency in culture, but primarily formed glia *in vivo* under steady-state conditions. However, these glial cells underwent neurogenesis in response to injury^7^,^10^. These studies were performed using genetic glial lineage tracing systems, in which transgenic animals with Sox10 and GFAP promoters were used as *Cre* drivers to identify glial lineages specifically. In this system, though Sox10-expressing cells usually lost their neurogenic potential in postnatal life, glial cells expressing Sox-10 were marked and they began to also express neuronal markers after BAC injury. These findings suggested that enteric glial cells have neurogenic potential *in vivo* and can generate enteric neurons in response to ENS injury^7^. In our study, we also observed many BrdU-positive glial cells after BAC injury, readily detectable in both wildtype and *W/W*^*v*^ mice (data not shown). This suggested that these newly generated glial cells were a potential source of ectopic neurons in response to injury and c-Kit suppression. It is noteworthy that at least 3 weeks after BAC treatment, distinct subsets of ectopic cells that were components of enteric ganglia were observed. These were GFAP-positive glia and cells positive for either excitatory or inhibitory neuronal markers. Given that ectopic neurons are derived from enteric glial precursor cells, the final differentiation from glia to neurons may have been completed by 3 weeks after BAC injury.

More recently, a subset of SCPs, which invade the gut alongside the extrinsic nerve, were shown to differentiate into specific neuronal subtypes during the postnatal period, suggesting SCPs may be another source of enteric neurons after birth^26^. Whether SCPs are involved in adult neurogenesis after injury remains unknown, but many SCPs can differentiate into specific neuronal types, including calretinin-positive excitatory motor neurons. The ectopic neurons observed in our study exhibited staining for other neuronal markers, unlike neurons differentiated from SCPs, and the newly appeared cells did not localize predominantly on the mesentery side. Therefore, it is unlikely that SCPs were the source of ectopic neurons in our study. However, our results were not sufficient to rule out the possibility of SCPs involvement.

One intriguing observation in this study was the appearance of ectopic neurons in the LM and SS, sites having no neurons in the normal gut. However, in most studies, new neurons in the adult appeared primarily in the MP^6^,^7^. Because most ectopic neurons were BrdU-positive, they were assumed to be newly divided neurons. In enteric neurons and precursor cells, 5-HT_4_-mediated signaling was reported to enhance neuronal survival, decrease apoptosis and promote neurogenesis, presumably via protein kinase A (PKA)- and phosphorylated cAMP response element-binding protein (pCREB)-dependent pathways^2^. These data suggested the existence of “germinal niches” between MP and LM layers and that progenitor cells migrated into myenteric ganglia in response to 5-HT_4_ stimulation^2^. Differentiation and migration of progenitor cells from the germinal niche required a relatively long time, 16–24 weeks, and these differentiated neurons did not appear to migrate to the LM. Therefore, the mechanism underlying ectopic neurogenesis demonstrated in our study may be distinct from these previously described 5-HT_4_-mediated mechanisms. However, further studies will be necessary to clarify this. Though we did not determine the exact source of neurons we observed, the ectopic localization of new neurons and glia in the LM and SS may be caused by chemo-attractive factors released from the tissue damaged by BAC.

As shown in Fig. 1, minor neurogenesis was detected in wildtype mice after BAC treatment, but neurogenesis in *W/W*^*v*^ mice was more marked. In addition, imatinib, which blocks c-Kit activity, enhanced neurogenesis after BAC treatment in wildtype mice. A possible explanation for induction of neurogenesis following suppression of c-Kit signaling is involvement of a neurogenesis suppressor affecting neuronal progenitor cells in a c-Kit-mediated manner. Because mast cells and ICC are the primary cells expressing c-Kit in the intestine, either cell type might continuously release an inhibitory mediator of neural precursor cells under steady-state conditions. Though there is no evidence for a neurogenesis suppressor, the participation of some inhibitory factors for neurogenesis *in vivo* was proposed by Joseph *et al.*^10^. These investigators demonstrated that adult enteric glia had the potential to form neurons in culture, but were fated to form glia *in vivo* both under normal physiological conditions and after injury^10^.

Though suppression of c-Kit signaling is crucial, it should be noted that this alone is insufficient to promote neurogenesis. A loss of neuronal components or damage is a basal factor promoting neurogenesis. This was supported by our findings that ectopic neurogenesis did not occur in either wildtype or *W/W*^*v*^ mice without neuron ablation or tissue damage by BAC, and that BAC treatment induced less neurogenesis in wildtype than in *W/W*^*v*^ mice.

In addition, the possibility remains that c-Kit downstream factors were involved. *W/W* mice, which were another mutant strain with a total loss of c-Kit signaling, had significantly decreased gene expression of msh-like 2 (*msx2*), a transcriptional factor, and neurotrophic tyrosine kinase receptor type 2 (*ntrk2*, *TrkB*), suggesting that these two genes were downstream of c-Kit signaling^27^. TrkB expression was observed in enteric glial cells, and its deficiency led to abnormalities in glial cells in ganglia, with no significant effect on neuron numbers^28^. These two reports suggested a lack of glial TrkB signaling following c-Kit suppression, though further studies are needed to support this interpretation. The possibility that glial cells initiated neurogenesis in the ENS following injury was also raised in one study^29^. In any event, both removal of neuronal components or injury, together with c-Kit suppression, may drive adult neurogenesis in the ENS.

## Methods

### Animal procedures

WBB6F1/Kit-*Kit*^*w*^*/Kit*^*w-v*^/Slc (*W/W*^*v*^) and C57BL/6NcrSlc mice (wildtype) mice at 5–7 weeks of age were purchased from Japan SLC (Hamamatsu, Japan). Animals were anesthetized with pentobarbital (45 mg/kg) via intraperitoneal injection. All procedures performed on laboratory animals were approved by the Institutional Animal Care and Use Committee of Nagoya University Graduate School of Medicine. All animal experiments were carried out in accordance with the Guidelines for Animal Experimentation of Nagoya University Graduate School of Medicine.

### BAC treatment

The abdomen was opened and the portion of ileum to be treated was wrapped for 30 min with a Kimwipe tissue (approximately 0.5-cm width), soaked in 0.05% benzalkonium chloride solution (Wako, Osaka, Japan), around the serosal surface of the distal ileum approximately 1.5 cm anterior to the caecum. To prevent adhesion of the operated area to the caecum, anti-adhesive solution (THN-01; Otsuka, Tokyo, Japan) was sprayed around the caecum. After treatment, the treated areas were rinsed with 0.1 M phosphate-buffered saline (PBS). Mice that were used for 5-bromo-2′- deoxyuridine (BrdU) analysis were supplied with drinking water containing 1 mg/mL BrdU (Sigma, Saint Louis, Missouri, USA). For imatinib administration, 2.5 μL/g imatinib solution (50 mg/mL in DW; Tokyo Kasei Kogyo Co, Tokyo, Japan) was administrated orally on alternate days.

### NADPH-diaphorase staining

The tissue used for analysis was removed from animals and balloon-like structures were made filled with 0.1 M PBS, tying the ends with thread. After brief fixation by immersion in 4% paraformaldehyde for 5 min and rinsing in 0.1 M PBS, the tissue samples were incubated for approximately 15–30 min in NADPH-diaphorase solution containing 0.1% β-NADPH (Oriental Yeast, Tokyo, Japan), 0.025% nitro blue tetrazolium (Wako) and 0.05% Triton-X in 0.01 M phosphate buffer (PB) in a 37 °C water bath. When staining for nerve elements was detected, reactions were stopped by removing samples from the solution. After complete fixation with 4% paraformaldehyde, the intestine was cut along mesenchymal sheets and the mucosa and circular muscle layers were peeled off under a dissection microscope. The whole-mount stretch preparations were covered with slide glasses. The specimens were observed under a light microscope (BX23; Olympus, Tokyo, Japan).

### Immunohistochemistry

Samples were fixed in Zamboni’s fixative containing 0.2% picric acid and 2% paraformaldehyde in 0.2 M PB overnight at 4 °C. To produce whole-mount stretch preparations, the specimens were rinsed in dimethyl sulfoxide for 30 min and then PBS. After peeling off the circular muscle layers, isolated longitudinal muscle layers attached to the myenteric plexus were placed in PBS containing 0.3% Triton-X for 20 min and then pre-incubated in 4% Block Ace solution (DS Pharma Biomedical, Osaka, Japan) for 20 min. The specimens were subsequently incubated with primary antibodies (shown in Table 1). Next, the specimens were rinsed and incubated with secondary antibodies conjugated to Alexa 488 (goat anti-rabbit IgG, goat anti-rat IgG, or donkey anti-goat IgG; 1:500; Invitrogen, Eugene, Oregon, USA) or Alexa 594 (goat anti-Rabbit IgG or donkey anti-Rabbit IgG; 1:500; Invitrogen). To detect nuclei, the specimens were also incubated with DAPI (1:1000, Dojindo, Kumamoto, Japan). The stained specimens were observed under a fluorescence microscope (BX23; Olympus; objective lens 10 ×/N.A. 0.40) with image acquisition by HCImage Live (Hamamatsu Photonics, Shizuoka, Japan) or were observed under a confocal laser scanning microscope (FLUOVIEW FV10i; Olympus; objective lens 10 ×/N.A. 0.40 or 60 ×/N.A. 13.5).

### Electron microscopy

The balloon-like structures, as described under the section on NADPH-diaphorase staining, were immersed in a fixative containing 1.5% glutaraldehyde and 4% paraformaldehyde in 0.1 M PB for 30 min at 4 °C. After rinsing in the same buffer, the specimens were incubated in NADPH-diaphorase solution containing 0.005% Triton-X for 15 min at 37 °C. After viewing the stained samples under the microscope, only certain segments were cut off and again fixed in the same fixative for 2 h at 4 °C. These specimens were post-fixed in 1% osmium tetroxide in the same buffer for 2 h at 4 °C, rinsed with distilled water, block-stained overnight in a saturated solution of uranyl acetate, dehydrated in ethyl alcohol series, and embedded in epoxy resin.

To detect NADPH-positive cells, 3-μm sections were cut using an ultramicrotome (UC7k; Leica Microsystems, Wetzlar, Germany) and only sections containing these cells were again embedded in epoxy resin on slide glasses. Ultrathin sections were then cut using the ultramicrotome. The sections were double-stained with uranyl acetate and lead citrate and processed for observation with a transmission electron microscope (JEM-1400Plus; JEOL, Tokyo, Japan).

### Statistical analysis

All values are expressed as means ± SEM. Results were statistically analyzed using the Mann–Whitney U test. A *p* < 0.05 was regarded as statistically significant.

## Additional Information

**How to cite this article**: Tamada, H. and Kiyama, H. Suppression of c-Kit signaling induces adult neurogenesis in the mouse intestine after myenteric plexus ablation with benzalkonium chloride. *Sci. Rep.* **6**, 32100; doi: 10.1038/srep32100 (2016).

## Figures and Tables

**Table 1:** List of primary antibodies used in this study.

Antigen	Host species	Type	Dilution	Company
PGP9.5	Rabbit	Polyclonal	1:500	Ultraclone (Yarmouth, UK)
Calretinin	Goat	Polyclonal	1:1000	Millipore (Temecula, CA, USA)
GFAP	Rabbit	Polyclonal	1:2 (FLEX Ready-to-use)	Dako (Glostrup, Denmark)
TH	Rabbit	Polyclonal	1:500	Millipore
BrdU	Rat	Monoclonal	1:200	AbD serotec (Oxford, UK)
